# Increased Neutrophil Count and Decreased Neutrophil CD15 Expression Correlate With TB Disease Severity and Treatment Response Irrespective of HIV Co-infection

**DOI:** 10.3389/fimmu.2020.01872

**Published:** 2020-08-28

**Authors:** Lerato N. Ndlovu, Lauren Peetluk, Sashen Moodley, Shepherd Nhamoyebonde, Abigail T. Ngoepe, Matilda Mazibuko, Khadija Khan, Farina Karim, Alexander S. Pym, Fernanda Maruri, Mahomed-Yunus S. Moosa, Yuri F. van der Heijden, Timothy R. Sterling, Alasdair Leslie

**Affiliations:** ^1^Africa Health Research Institute, University of KwaZulu-Natal, Durban, South Africa; ^2^Department of Infectious Diseases, University of KwaZulu-Natal, Durban, South Africa; ^3^Vanderbilt Tuberculosis Center, Vanderbilt University School of Medicine, Nashville, TN, United States; ^4^Global Division, The Aurum Institute, Johannesburg, South Africa; ^5^Department of Infection and Immunity, University College London, London, United Kingdom

**Keywords:** tuberculosis biomarker, neutrophil phenotype, blood neutrophil count, tuberculosis treatment outcome, tuberculosis disease severity

## Abstract

Tuberculosis remains a leading cause of death globally despite curative treatment, partly due to the difficulty of identifying patients who will not respond to therapy. Simple host biomarkers that correlate with response to drug treatment would facilitate improvement in outcomes and the evaluation of novel therapies. In a prospective longitudinal cohort study, we evaluated neutrophil count and phenotype at baseline, as well as during TB treatment in 79 patients [50 (63%) HIV-positive] with microbiologically confirmed drug susceptible TB undergoing standard treatment. At time of diagnosis, blood neutrophils were highly expanded and surface expression of the neutrophil marker CD15 greatly reduced compared to controls. Both measures changed rapidly with the commencement of drug treatment and returned to levels seen in healthy control by treatment completion. Additionally, at the time of diagnosis, high neutrophil count, and low CD15 expression was associated with higher sputum bacterial load and more severe lung damage on chest x-ray, two clinically relevant markers of disease severity. Furthermore, CD15 expression level at diagnosis was associated with TB culture conversion after 2 months of therapy (OR: 0.14, 95% CI: 0.02, 0.89), a standard measure of early TB treatment success. Importantly, our data was not significantly impacted by HIV co-infection. These data suggest that blood neutrophil metrics could potentially be exploited to develop a simple and rapid test to help determine TB disease severity, monitor drug treatment response, and identify subjects at diagnosis who may respond poorly to treatment.

## Introduction

Though global tuberculosis (TB) disease incidence is falling at a rate of 1.5–2% per year, it remains one of the leading causes of death from a single infectious agent, and much greater declines in incidence are required to meet global TB targets (1). TB is a curable disease, but better tools to evaluate early TB treatment response would facilitate optimization of TB treatment, and cure rates (2, 3). Shorter TB treatment regimens are also needed as poor adherence to the current standard 6-months regimen can lead to treatment failure, relapse, or the development of drug resistant TB (4). Likewise, tools for identifying patients at the point of diagnosis who are at risk of responding poorly to treatment could help with clinical management. In addition, novel methods of evaluating response to treatment could expedite testing of new TB treatment regimens in clinical trials. Host immune responses are potential biomarkers for predicting response to anti-TB treatment (5, 6) as the current approach of evaluating culture conversion at 2 months has proved poorly predictive of cure (7–9). Typically, these biomarkers are measured in blood or urine, which also makes them an attractive option for groups that are inherently difficult to diagnose and monitor with conventional sputum samples, such as children, pregnant women, and people living with HIV (10–12).

As proof of this concept, Adekambi et al. showed that the expression of cellular activation markers on *Mycobacterium tuberculosis* (Mtb)-specific CD4+ T-cells was highly effective at distinguishing individuals with asymptomatic latent TB infection (LTBI) from those with untreated active TB (13). Furthermore, decreasing expression of these markers correlated with decreasing mycobacterial load during treatment. These observations were subsequently confirmed in HIV/TB co-infected individuals (14). The advantage of this biomarker is that it is highly TB disease specific. However, the assay requires an extended period of cell stimulation and also, by definition, is only useful in individuals with detectable Mtb responses. This last point is important, as up to 30% of people with active TB infection do not have a detectable Mtb-specific CD4 response at the time of diagnosis (15), and this proportion can be higher in individuals co-infected with HIV (16, 17).

Neutrophils are the most abundant immune cells in the blood, accounting for ~60% of circulating leukocytes, and are rapidly activated and recruited to sites of infection or tissue damage (18). Moreover, neutrophils have a lifespan in circulation of ~7 h, increasing on activation to 2–4 days. Consequently, the large pool of neutrophils in blood and tissue is continuously being replenished from precursors in the bone marrow (19–21). Therefore, neutrophil based biosignatures may be expected to change rapidly. The role neutrophils play during active TB is complex and depends on timing. Initially, following arrival at the site of infection, neutrophils can recognize Mtb through both opsonin-dependent and -independent pathways, resulting in phagocytosis (22, 23). This probably contributes to early immune control, as experimental depletion of neutrophils during initial infection in mice has a negative outcome for the host (24). In addition, low blood neutrophil count in household contacts of TB patients is an independent predictor of TB infection (25). However, if infection fails to resolve, neutrophils appear to be one of the main drivers of lung destruction (6, 26, 27). A detailed investigation of the neutrophil response to TB infection in humans may also, therefore, improve our understanding of the immunopathology of this disease.

Transcriptomic analysis of whole-blood from TB patients has revealed a 393-transcript signature for active TB disease that is dominated by a neutrophil-driven IFN-inducible gene profile, consisting of both IFN-γ and type I IFN-αβ signaling (28). This signature was subsequently confirmed in sorted neutrophils and found to correlate with radiographic extent of disease and could distinguish active TB disease from other respiratory diseases. In addition, this signature significantly diminished following 2 months of TB treatment (29). We, therefore, hypothesized that (1) active TB is associated with both increased blood neutrophil count and phenotypic changes that could be easily measured by flow cytometry, (2) neutrophil count and phenotype is correlated with disease severity and, (3) due to the lifespan of neutrophils, these changes rapidly respond to changing disease status.

## Materials and Methods

### Study Population

#### TB Cases

Study participants were recruited from the Regional Prospective Observational Research for Tuberculosis (RePORT)-South Africa, Durban study between December 2016 and December 2019. All participants were TB symptomatic and referred for TB testing by attending clinician. Enrolled subjects were GeneXpert and culture positive (solid or liquid media), had evidence of pulmonary involvement by chest X-ray (CXR), were 18 years or older and provided written informed consent. All RePORT study participants received standard 6-months treatment for TB, consisting of isoniazid, rifampicin, pyrazinamide, and ethambutol. Study participants were followed for the duration of TB treatment and up to 24 months post-enrolment. Enrolees found to have baseline drug resistance to any standard anti-TB drug (isoniazid, rifampicin, ethambutol, or pyrazinamide), who developed resistance during treatment, or who had concomitant extrapulmonary TB were excluded from the study.

#### Controls

TB symptomatic participants initially recruited for RePORT as GeneXpert positive, but subsequently found to be culture negative at baseline were included as Xpert-positive/culture-negative controls (*n* = 14) and were followed for 2 months from enrolment. An additional control group consisting of healthy asymptomatic individuals (*n* = 23) were recruited from the Collection of Urine, Blood, and Sputum (CUBS) cohort in Durban between January and December 2018. This group is referred to as healthy controls.

#### Descriptive Statistics

For TB cases and Xpert-positive/culture-negative controls age, sex, race, body mass index (BMI), self-reported alcohol use, tobacco use, and employment status were recorded. For persons living with HIV (PLWH), CD4 cell counts, HIV-1 RNA (viral load), and timing of antiretroviral therapy were also collected. Extent of lung disease at enrolment was determined from CXR, evaluated by a radiologist blinded to all study participant information, using a CXR score [% area of lung infected + 40 for the presence of cavities; giving a scale of 0–140 (30)] see Table S1. Bacterial burden was estimated from liquid culture using the BD BACTEC MGIT 960 mycobacterial detection system (Becton Dickinson Microbiology Systems, Sparks, Md.) and full blood counts were measured independently by a commercial laboratory. For the healthy controls age, sex, and race were recorded.

### Sample Collection, Processing, and Analysis

#### Samples

Blood samples were collected from healthy controls at one time-point only, and blood and sputum samples from TB cases were collected at baseline (pre-treatment) and at post-treatment initiation study visits: week 1, week 3, month 2, and month 6. For all participants, fresh blood samples were collected in EDTA vacutainers (BD Biosciences) for cell phenotyping.

#### Neutrophil Isolation and Staining

A 100 μl of whole blood was stained with 13 surface markers and incubated for 30 min at 4°C in the dark. The phenotyping panel comprised of standard lineage markers V500 conjugated mAb anti-CD45 (clone HI30), BUV496 conjugated mAb anti-CD16 (clone 3G8), BUV395 conjugated mAb anti-CD11b (clone ICRF44), V450 conjugated mAb anti-CD66b (clone G10F5) all from BD Bioscience; BUV785 conjugated mAb anti-CD3 (clone OKT3) and BV711 conjugated mAb anti-CD14 (clone M5E2) from Biolegend. The following phenotype markers were used, PE conjugated mAb anti-CD177 (clone MEM-166) and BV605 conjugated mAb anti-HLA-DR (clone G46-6) from BD Bioscience; FITC conjugated mAb anti-CD15 (clone HI98) and PE-Cy7 conjugated mAb anti-CXCR4 (clone 12G5) from Biolegend; APC conjugated mAb anti-CD32 (clone FLI8.28) and PE-Cy5 conjugated mAb anti-CD10 (clone HI10a) from BD Pharmigen and Live dead stain (ThermoFischer Scientific). Phenotype markers were chosen based on preliminary data from our lab indicating differential expression between TB cases and HIV-TB- controls. Red blood cells were removed, and leukocytes fixed by adding 2 mls of FacsLyse solution (BD Bioscience) and incubating for 10 min in the dark at room temperature. Samples were then centrifuged at 500 g for 5 min and the supernatant decanted. Cells were washed with 2 mls of 1X PBS (Sigma Aldrich) for 5 min at 500 g and the cell pellet resuspended in 2% PFA (Sigma Aldrich). Cells were acquired on a 17 color FACSAria Fusion (BD Bioscience), with calibrating beads run before each acquisition to ensure consistent performance. Due to the longitudinal nature of the study we normalized the data based on a published method by Upreti et al. (31). Data normalization was performed by transforming the Mean Fluorescence Intensity (MFI) values of the test samples (TB cases, Xpert-positive/culture-negative, and healthy controls) to a common scale using the following equation: Final relative fluorescence intensity (RFI) = MFI of the test sample/MFI of the internal control. Gating strategy shown in Figure S1.

#### TB Treatment Outcomes

We examined two TB treatment outcomes; (1) culture status on solid media 2 months after initiation of anti-TB therapy; and (2) end of TB treatment outcome, as defined by World Health Organization (WHO) into two mutually-exclusive categories—treatment success (cure or treatment completion) vs. unsuccessful outcome (death, failure, loss to follow-up, or not evaluated) (32). Cure was defined as completing treatment with a negative culture result at the end of treatment and on at least one previous occasion. Treatment completion was based on completion of standard anti-TB therapy without evidence of failure, and without documentation of a negative culture in the last month of TB treatment and/or on at least one previous occasion (either because it was not done, or results are unavailable). Death included mortality from any cause during TB treatment. Treatment failure was defined as a positive culture at month 5 of treatment or later. Loss to follow-up was any participant who interrupted treatment for 2 consecutive months or more. Not evaluated included any participant for whom treatment outcome was unassigned, including cases who transferred out of care or whom treatment outcome was unknown.

In addition, for exploratory analyses, we categorized TB cases into four groups based on time to culture negativity. Individuals who were culture negative within 1 month of treatment initiation were defined as early responders, those who converted to culture-negative by 2 months as medium responders, those who converted to culture-negative at 6 months were slow responders, and treatment failures were those who remained culture positive during the full 6 months of treatment.

### Statistical Analysis

Baseline characteristics were compared between TB cases, Xpert-positive/culture-negative, and healthy controls using Chi-square or Fisher's exact test for categorical variables and Kruskal-Wallis test for continuous variable. Correlations between CXR score, bacterial burden, blood counts, neutrophil phenotype, and demographic data were determined using the Mann-Whitney test and Spearman's correlation analysis. Differences in distributions and median values of neutrophil blood counts and CD15 expression were compared between cases and controls and also examined for changes from baseline during the course of TB treatment using the Wilcoxon rank sum test and Kruskal-Wallis test followed by Dunn's multiple comparison test. We graphically evaluated the impact of pre-treatment immune measures on patient response to treatment, defined by early, medium, slow and treatment failure responses. Additionally, we examined the association between baseline immune markers and culture status at month 2 (positive vs. negative) and end of treatment outcome (successful vs. unsuccessful treatment) using univariable and HIV-adjusted logistic regression. Confounders, including age, sex, baseline BMI, and HIV were considered *a priori* based on clinical expertise. Due to a limited number of outcome events, adjusted models controlled only for HIV co-infection, as it was believed to be most clinically relevant confounder. We also examined the association between the aforementioned exposures with treatment failure/death and with loss to follow-up/not evaluated. The referent group for these analyses was successful outcome (i.e., in analyses with the outcome of failure/death), persons who were lost to follow-up/not evaluated were excluded, and in analysis with the outcome of lost to follow-up/not evaluated, persons with failure/death were excluded. Statistical analysis was performed using StataIC, version 16 (StataCorp, College Station, TX, USA) and GraphPad PRISM, version 8.3.1 (GraphPad Software, San Diego, CA, USA).

## Results

The characteristics of the study population are listed in Table 1. Of note, HIV co-infection was 63%, the majority of whom had not started antiretroviral therapy (ART) at baseline (67%). As expected, smoking was significantly higher in the TB subjects (22%) compared to healthy controls (4%). HIV co-infected individuals had significantly lower CXR scores, primarily due to fewer cavities (*P* = 0.0028); they also had lower bacterial burden than HIV uninfected individuals (median HIV- = 34.54 and HIV+ = 31.88; *P* = 0.0083) (Figure S2A). Consistent with these results, we observed a weak correlation between CD4 count and CXR score (*r* = 0.2370; *P* = 0.0268) and bacterial load (*r* = 0.2478; *P* = 0.0143, Figures S2D,E).

**Table 1:** Baseline clinical and demographic characteristics of culture positive, drug susceptible TB cases; Xpert-positive/culture-negative controls and healthy controls.

**Characteristics *N* (%) or Median [IQR]**	**TB cases (*n* = 79)**	**Xpert-positive/culture-negative controls (*n* = 15)**	**Healthy controls (HC) (*n* = 23)**	***P*-value^§^**
Age	34 (7–43)	37 (30–40)	32 (25–38)	0.37
Male sex	53 (67)	9 (60)	12 (52)	0.41
HIV status (positive)	50 (63)	11 (73)	0 (0)	<0.01^*^
On antiretroviral therapy	18 (23)	6 (40)	-	0.16
CD4 count	350 [156–608]	386 [111–536]	-	0.87
Current smoker	17 (22)	4 (27)	1 (4)	<0.01^*^
BMI (kg/m^2^)	20.7 [18.6–22.8]	20.2 [19.0–22.4]	-	0.90
Employed	35 (44)	7 (47)	-	0.87
Previous TB	16 (20)	5 (33)	-	0.26
Bacterial load (MGIT^a^)	32.8 [28.7–35.3]	-	-	-
Cavitation present	55 (74)	3 (25)	-	<0.01^*^
Chest-X ray score (CXR)	70 (35–80)	8.5 [3.5–56]	-	0.02^*^

§ *P-values determined by Fisher's exact, chi square, or Wilcoxon rank sum tests*.

* *P < 0.05*.

a *MGIT, Mycobacteria Growth Indicator Tube*.

*Values are bold to highlight statistical significance compared to the other values*.

### Neutrophil Blood Count and CD15 Expression Level Are Altered at Baseline and Associated With Disease Severity

**Figure 1:**
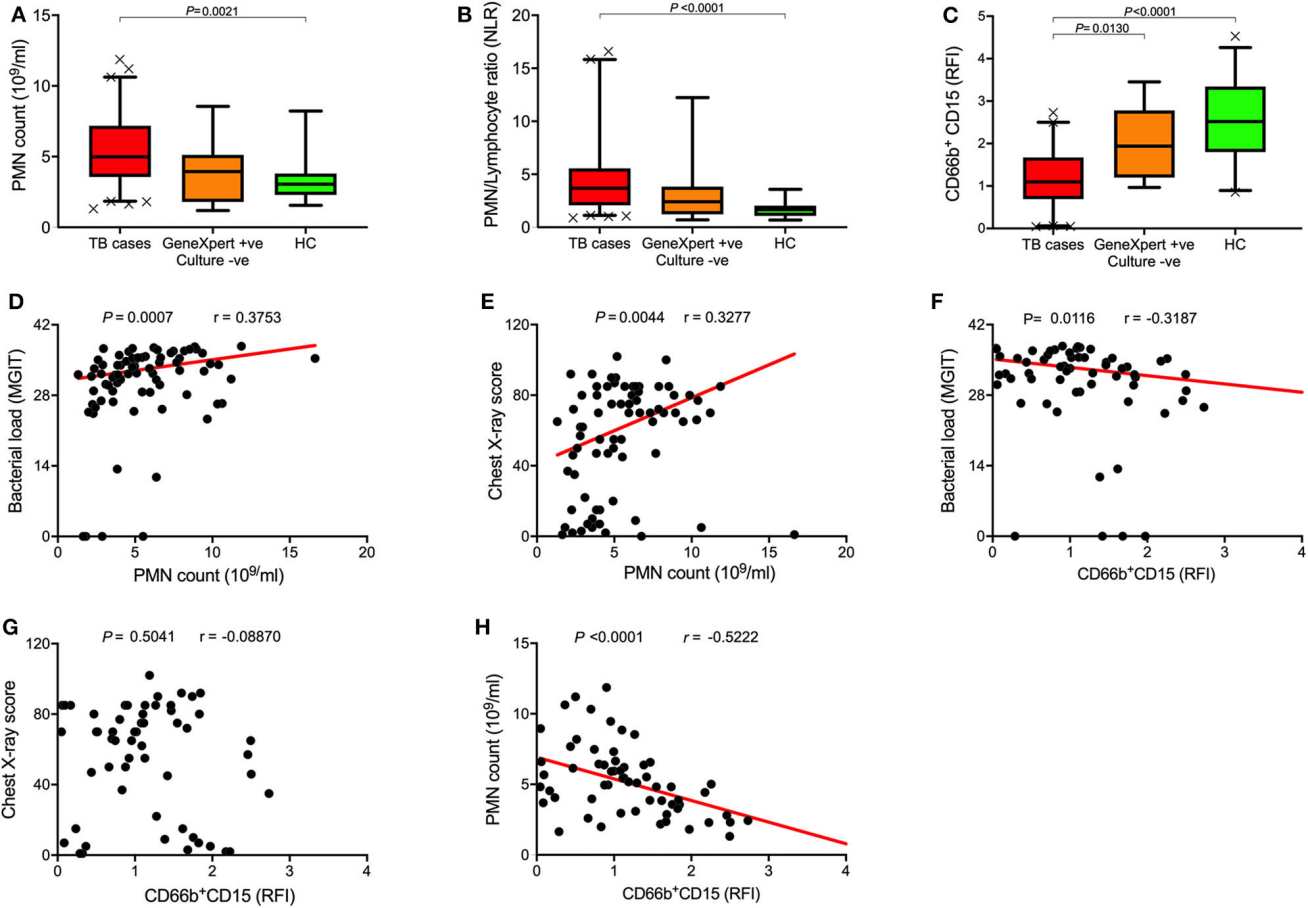
Baseline neutrophil blood counts and phenotypic changes in active TB disease. **(A–C)** Box plots of **(A)** baseline neutrophil blood count (PMN count), **(B)** PMN/Lymphocyte ratios, and **(C)** Surface CD15 expression levels of TB cases (*n* = 79), Xpert-positive/culture-negative controls (*n* = 15), and healthy controls (HC; *n* = 23), Data represents median values and was analyzed using Kruskal-Wallis test for Dunn's multiple comparisons. **(D–H)** Spearman's rank correlation analysis of neutrophil blood counts and CD15 expression with bacterial burden and chest X-ray score.

Full blood counts were measured at baseline and during the course of treatment. Neutrophil count and neutrophil/lymphocyte ratio (NLR) were significantly higher in TB cases at diagnosis compared to healthy controls (HC) (Figures 1A,B). Interestingly, symptomatic Xpert-positive/culture-negative controls also had slightly higher neutrophil counts and NLR compared to healthy controls, but this did not reach statistical significance. Of the surface markers measured in this study, CD15 expression level showed the most consistent difference between TB cases and controls at baseline. As shown in Figure 1C, surface expression of CD15, a canonical neutrophil marker, was found to be highly down regulated at baseline in TB patients compared to healthy controls (*P* < 0.0001). In addition, TB symptomatic Xpert-positive/culture-negative controls displayed significantly higher CD15 expression levels than active TB cases (*P* = 0.01).

To explore the relationship between baseline neutrophils and disease severity, we performed univariate correlations with sputum bacterial load and CXR score, clinically important measures of TB disease severity. Despite the relatively small sample size, we observed significant correlations between neutrophil count and both bacterial load (*r* = 0.25, *P* = 0.034; Figure 1D) and CXR score (*r* = 0.41, *P* = 0.0004; Figure 1E), and CD15 expression and bacterial load (*r* = −0.3187; *P* = 0.01; Figure 1F). Together these data support the existence of a relationship between the blood neutrophil response to TB and disease pathogenesis in the lung, as suggested by several recent publications (6, 33, 34). Finally, we found CD15 expression level was inversely correlated with neutrophil count (*r* = −0.5222, *P* < 0.0001; Figure 1H) but had no correlation with CXR score (Figure 1G). Importantly these measurements were taken independently by our in-house flow cytometry assay and by an accredited commercial laboratory, thereby adding support to the observed phenotypic changes.

### Neutrophil Count and CD15 Expression Resolved With TB Treatment

**Figure 2:**
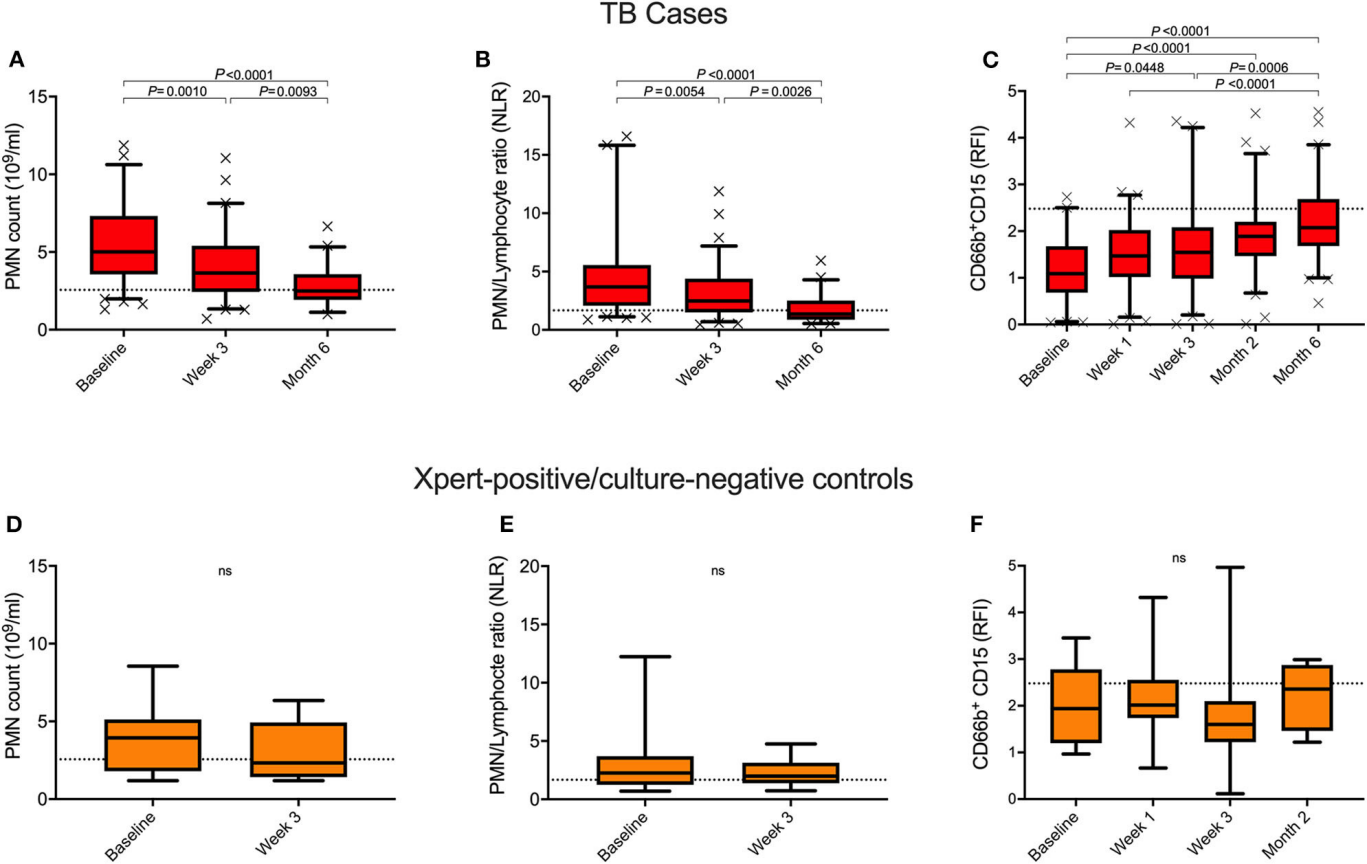
Longitudinal measurement of blood neutrophil count and CD15 expression during anti-TB treatment. Changes in neutrophil blood count (PMN), PMN/Lymphocyte ratio and surface CD15 expression levels over the course of treatment in TB cases **(A–C)** and Xpert-positive/culture-negative controls **(D–F)**. *P*-values were obtained using the Wilcoxon rank sum test and Kruskal-Wallis tests for Dunn's multiple comparisons. Dotted line represents the median healthy control levels.

**Figure 3:**
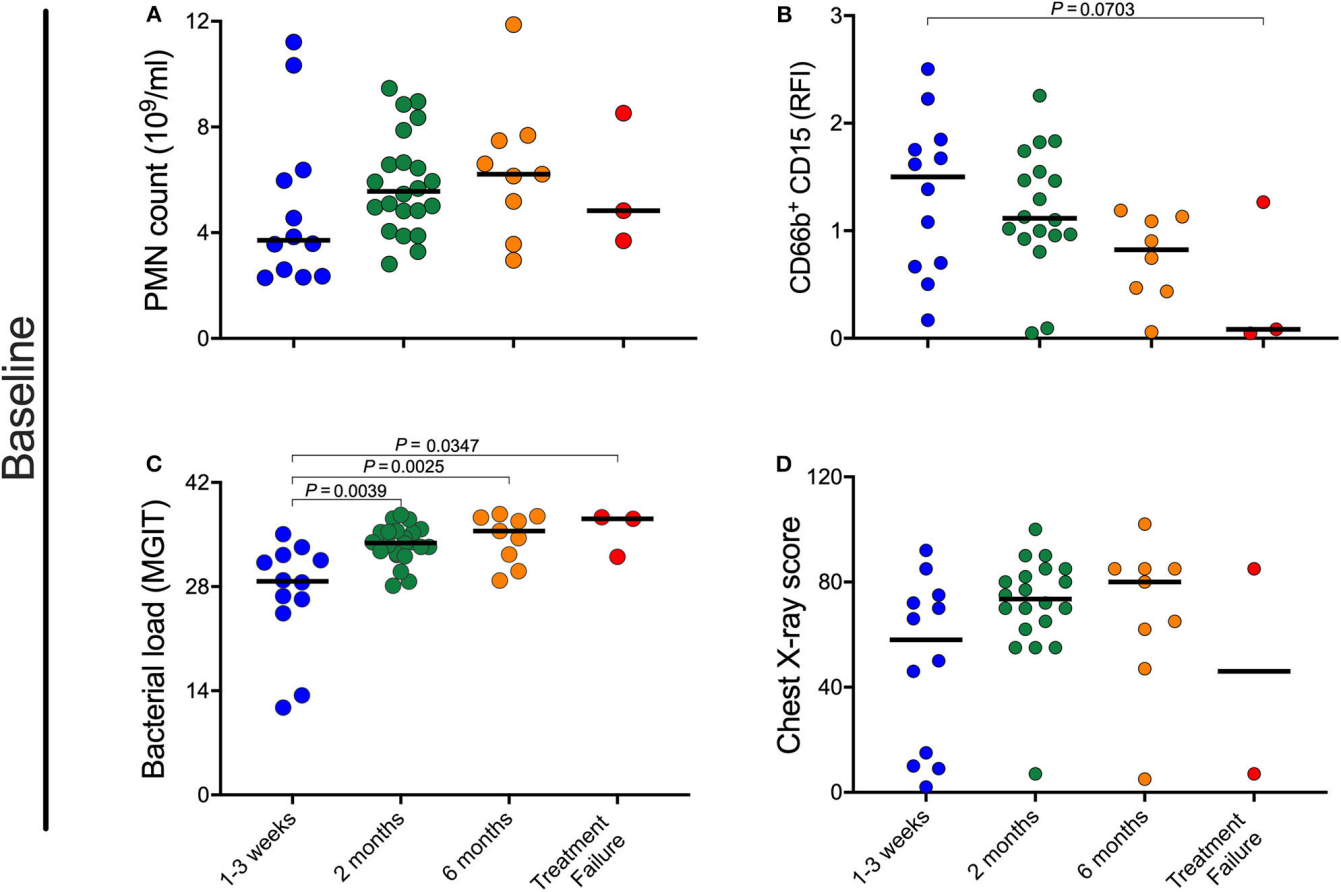
Impact of pre-treatment characteristics on rate of culture conversion. **(A–D)** Baseline neutrophil blood (PMN) count, CD15 expression, bacterial load, and chest X-ray scores were retrospectively separated into four groups based on time to MGIT culture negativity. Early responders became and remained culture negative within the 1st month, medium responders by 2 months, and slow responders by month 6 post-treatment initiation. In this analysis treatment failure are individuals who were culture positive at all time points. *P*-values were obtained using the Mann-Whitney test.

Neutrophil characteristics were examined longitudinally to determine how they respond to TB drug treatment. Baseline neutrophil count and NLR both significantly improved within the 1st month of TB treatment, and reached the level seen in healthy controls by month 6 (Figures 2A,B), irrespective of HIV status (Figures 3A,B). Additionally, surface expression of CD15 increased during TB treatment and expression level was no longer significantly different from healthy controls by 2 months of treatment (Figure 2C and Figure S1). CD15 expression was already elevated by week 1 (for which complete blood counts were not available). This supports our hypothesis that rapid turnover of blood neutrophils makes them extremely sensitive to changes in the lung environment. This is also supported by the fact that, following 3 weeks of drug treatment, neutrophil count and CD15 expression remain correlated with falling sputum bacterial load (*r* = 0.3929, *P* = 0.0006 and *r* = −0.3498, *P* = 0.0046, respectively; median bacterial load baseline vs. month 1 = 33 vs. 21 *P* < 0.0001), and each other (*r* = −0.5751, *P* < 0.0001) (Figures S4A–C). As with neutrophil count, changes in CD15 expression were not affected by HIV co-infection (Figure S3C). Interestingly, no changes in neutrophil count, NLR and CD15 expression were detected among the Xpert positive/culture negative controls following 2 months of treatment with anti-TB drugs (Figures 2D–F).

### Pre-treatment Characteristics Are Associated With Rate of Culture Conversion

Given the correlation between neutrophil characteristics and clinical measures of disease severity at baseline, we sought to determine whether the baseline neutrophil characteristics, bacterial load, or chest X-ray changes correlated with treatment response kinetics. Initially, participants were grouped as being early, medium or slow responders as well as treatment failure, based on achieving and maintaining TB culture negative status at month 1, month 2, month 6, or never, respectively. As shown in Figure 3, there is a trend for individuals with a lower blood neutrophil counts and higher CD15 surface expression at baseline to become culture negative early compared to those who culture converted later or not at all (Figures 3A,B). These differences did not reach statistical significance by univariate analysis. Bacterial load at baseline, however, was significantly associated with treatment response, with early responders having significantly lower bacterial burden than all others (Figure 3C). CXR score, on the other hand, was not predictive of treatment response (Figure 3D).

Next, we examined the association between baseline neutrophil characteristics, CXR score, and bacterial load with sputum culture conversion at 2 months as an endpoint, a clinically relevant measure of TB treatment response (35). For this analysis, we excluded four participants whose baseline culture was positive on liquid media, but not solid media, and 8 participants who were missing culture results at month 2. Among 67 remaining participants with a solid culture at baseline, 7 (10%) remained culture positive after 2 months of anti-TB treatment. Results of this analysis are shown in Table 2. Interestingly, in this reduced dataset, the only baseline characteristic associated with remaining culture positive at month 2 was CD15 expression level (Odds Ratio (OR): 0.17, 95% Confidence Interval (CI): 0.03, 0.97). In other words, for every unit increase in baseline CD15, the odds of remaining culture positive after 2 months of TB treatment was reduced by 83%. After adjusting for HIV-status, the relationship appears slightly stronger (adjusted OR: 0.14, 95% CI: 0.02, 0.89).

**Table 2:** Crude and HIV-adjusted effect measures for association between baseline bacterial load, chest x-ray score, neutrophil (PMN) count, and CD15 with TB treatment outcomes (M2 culture conversion, unsuccessful outcome, death/failure, and losses to follow-up/not evaluated), using solid culture.

**Exposure**	**Outcome**	**Events/*N* (%)^a^**	**Unadjusted OR 95% CI)**	**HIV adjusted OR (95% CI)**
Bacterial load	M2 culture positive	7/67 (10%)	0.98 (0.90, 1.07)	0.98 (0.90, 1.06)
	Unsuccessful treatment	20/75 (27%)	0.99 (0.93, 1.06)	0.99 (0.93, 1.06)
	Death/failure	4/59 (7%)	0.98 (0.87, 1.10)	0.98 (0.85, 1.13)
	LTF/NE	16/71 (23%)	0.99 (0.92, 1.07)	0.99 (0.92, 1.06)
CXR score^b^	M2 culture positive	6/64 (9%)	1.02 (0.98, 1.06)	1.03 (0.98, 1.07)
	Unsuccessful treatment	18/71 (23%)	1.00 (0.98, 1.02)	1.00 (0.98, 1.02)
	Death/failure	3/56 (5%)	0.99 (0.96, 1.03)	1.00 (0.96, 1.04)
	LTF/NE	15/68 (22%)	1.00 (0.98, 1.02)	1.00 (0.98, 1.02)
PMN count^c^	M2 culture positive	7/67 (10%)	1.06 (0.82, 1.37)	1.06 (0.81, 1.37)
	Unsuccessful treatment	19/74 (26%)	0.90 (0.73, 1.11)	0.90 (0.73, 1.11)
	Death/failure	3/58 (5%)	0.68 (0.35, 1.30)	0.79 (0.44, 1.40)
	LTF/NE	16/71 (23%)	0.94 (0.76, 1.15)	0.92 (0.74, 1.15)
CD15^d^	M2 culture positive	6/56 (11%)	**0.17 (0.03, 0.97)**	**0.14 (0.02, 0.89)**
	Unsuccessful treatment	10/58 (17%)	0.57 (0.21, 1.57)	0.58 (0.21, 1.58)
	Death/failure	3/51 (6%)	0.54 (0.09, 3.21)	0.68 (0.15, 3.09)
	LTF/NE	7/55 (13%)	0.58 (0.18, 1.87)	0.57 (0.17, 1.89)

a *Complete case analysis. The referent group for M2 culture positive is M2 culture negative (n = 8 people missing culture results at M2 were excluded). The referent group for unsuccessful outcome, death/failure, and loss to follow-up/not evaluated was successful outcome. In analyses with the outcome of failure/death, persons who were lost to follow-up/not evaluated were excluded, and in analysis with the outcome of lost to follow-up/not evaluated, persons who with failure/death were excluded*.

b *Missing: n = 4*.

c *Missing: n = 1*.

d *Missing: n = 17*.

*Values are bold to highlight statistical significance compared to the other values*.

### TB Treatment Outcome

We also examined the association between bacterial load, CXR score, neutrophil count, and CD15 with TB treatment outcome, defined according to the WHO definitions. Among 75 TB cases who were positive for Mtb on solid culture, 53 (71%) were cured, 2 (3%) completed treatment, 2 (3%) failed treatment), 2 died (3%), 8 (11%) were lost to follow-up (LTF), and 8 (11%) were not evaluated (NE). Combining death, failure, loss to follow-up, and not evaluated, the unsuccessful outcome rate was 27%. However, none of baseline bacterial load, CXR score, neutrophil count, or CD15 were associated with TB treatment outcomes (Table 2).

## Discussion

In this study we investigated changes in the frequency and phenotype of blood neutrophils associated with active TB at diagnosis and patient response to standard TB drug therapy. Overall, we find that active TB is associated with both an increase in neutrophil count and down-regulation of the canonical neutrophil marker CD15, irrespective of HIV co-infection. In addition, changes in neutrophil count and CD15 expression correlate with clinical markers of disease severity—sputum bacterial load, as measured by time to positivity in liquid culture, and extent of lung involvement, as determined by an independent radiology examination of the CXR. This suggests a potential direct relationship between the blood neutrophil response and the severity of lung disease. As neutrophils are short-lived and rapidly recruited to the site of infection, we hypothesized that any blood neutrophil signature would respond rapidly to clearance of the bacteria. Our data support that hypothesis, as both neutrophil count and CD15 expression level significantly increased by month 2. Moreover, CD15 expression, which was measured more frequently, was higher after only 1 week of drug treatment. The fact that the correlation between neutrophils and bacterial load is maintained after 1 month of treatment, when bacterial load has greatly reduced, also highlights the direct relationship between the blood neutrophil response and the kinetics of the response to drug therapy. Finally, in a multivariate analysis, baseline CD15 expression level was the only variable that predicted sputum culture conversion at 2 months.

The potential role of neutrophils in the immune response to TB is somewhat conflicting and has been well-reviewed elsewhere (36, 37). However, targeting these cells as potential biomarkers for diagnosing active TB disease and monitoring treatment outcomes has been less well-studied (38, 39). Abakay et al. found that an elevated neutrophil count and NLR were both associated with advanced TB disease; with increased NLR, in particular, associated with the most severe lung involvement (40). Disease severity was estimated from CXR and thus our findings are comparable, although in our study, NLR was less informative than neutrophil count alone or CD15 expression level. Furthermore, our data are consistent with clinical studies and experimental infections showing that acute pulmonary tuberculosis is accompanied by an influx of neutrophils (26, 41). In mice, for example, neutrophil influx is associated with mycobacterial burden and lung pathology, and their depletion in chronic infection leads to decreased CFU and improved survival (26, 42–44). It is important to note, however, that the influx of neutrophils is not unique to TB disease and has been well-described in other inflammatory conditions such as asthma, influenza and bacterial or viral pneumonia (45–47). Therefore, neutrophil characteristics alone would not be appropriate as a diagnostic test. None the less, studies have shown the usefulness of neutrophil phenotyping in distinguishing bacterial infection from other inflammatory conditions (48, 49). Therefore, together with other diagnostic tests, neutrophil monitoring could be valuable as a rule our test and for identifying patients at base line with the most severe disease. Such metrics could prove useful for testing treatment shortening interventions, for example.

Unlike other immune subsets, it is not possible to freeze neutrophils for future analysis. Consequently, few studies have examined neutrophil phenotypic changes in human TB cohorts. The phenotyping of blood neutrophils by flow cytometry in this study was undertaken with the aim of potentially identifying sensitive markers of the neutrophil response. Initially, this included eight neutrophil surface markers based on preliminary data from our lab showing differential expression between healthy individuals and TB cases. However, in this longitudinal study, only CD15 expression showed a consistent response during the course of TB treatment. CD15 is a carbohydrate antigen associated with cell surface of glycoproteins or glycolipids, including CD11b/CD18 (Mac-1) and CR3 β-chain (50, 51). It is highly expressed by mature neutrophils (52) and has been shown to affect a number of neutrophil functions including adhesion to endothelium, phagocytosis, degranulation, and the respiratory burst (50, 53–55). However, there are very few studies that have examined CD15 expression level *ex vivo*. CD15 expression is elevated on low density neutrophils (56), which can arise from activation both *in vitro* and *in vivo* (57). Conversely, expression of CD15 was found to dramatically decreases in asthmatic patients following acute allergen challenge (58), suggesting that activation *per se* does not elevate CD15 in general. Interestingly, treatment of healthy donors with Granulocyte-Colony Stimulating Factor (G-CSF) to induce neutrophil release from the bone marrow (59, 60), also leads to decreased CD15 expression on blood neutrophils (61). Therefore, both the elevated blood neutrophil numbers and low CD15 expression observed in active TB may relate to sustained recruitment of neutrophils from the bone marrow in response to cytokine release from Mtb infection. As neutrophils mature in the bone marrow they upregulate CD15 (62, 63), implying that the CD15 low neutrophils we observed in blood are not fully mature. In addition, when G-CSF treatment was ceased in the above studies, CD15 expression level in returned to normal, consistent with the rapid response kinetics observed in our study. Whether the influx of CD15 low neutrophils observed in TB directly relates to G-CSF, alone or in combination with other neutrophil related cytokines, is an interesting area for future study and might lead to alternative and potentially more sensitive measures of on-going lung involvement.

In the analysis we included individuals who were TB symptomatic and GeneXpert positive at diagnosis, but turned out to be culture negative. Discordant gene-expert and culture results have previously been linked, at least in part, to residual DNA in the lung from killed bacteria (64), poor sputum quality and low bacterial load as determined by CT cycle number (65). One third of these subjects reported previous TB episodes, slightly higher than the culture positive group (20%), but detail on sputum quality and GeneXpert quantitation data was not recorded. Nonetheless, the neutrophil data on this group were interesting and potentially informative. First, neutrophil counts and CD15 expression in this group were not significantly higher than healthy controls, and, second, neither neutrophil count nor CD15 expression level were changed by 2 months of TB treatment. This suggests that monitoring blood neutrophils during TB screening could help to inform classification and management of discordant subjects. In addition, to identify novel prognostic factors, we sought to determine whether neutrophil characteristics at TB diagnosis were predictive of response to treatment. We found that baseline CD15 expression level was predictive of culture positivity at month 2, suggesting potential value in helping triage subjects into those likely to respond well to treatment. In the univariate analysis, sputum bacterial load also appeared to distinguish subjects who became culture negative rapidly compared to those with a slower response to treatment, consistent with other studies (66, 67). However, neutrophil phenotype can be determined much faster than sputum bacterial load, and thus could be more relevant in the clinical setting.

There were some limitations of this study. First, sample size was relatively small. However, it was sufficiently large to detect significant correlations between neutrophil count, CD15 expression level, and TB treatment outcomes. Second, study participants were from one city in South Africa, and may not be generalizable to other settings. However, there was a high proportion of HIV-positive participants, and we were able to demonstrate that findings did not differ according to HIV status. Further work is planned to further explore the potential utility of neutrophil signatures in larger cohorts and with additional measures of neutrophil activity and neutrophil related cytokines.

## Data Availability Statement

All datasets generated for this study are included in the article/Supplementary Material.

## Ethics Statement

The studies involving human participants were reviewed and approved by Biomedical Research Ethics Committee of the University of Kwa-Zulu Natal. The patients/participants provided their written informed consent to participate in this study.

## Author Contributions

AL and LN conceived, designed the experiments, and wrote the paper. AP, TS, FK, and M-YM designed and managed the cohort. FK and MM enrolled patients and collected samples and data. KK and SM supervised laboratory collection of clinical samples and analysis. LN, SM, SN, and AN performed the experiments. TS, YH, FM, and LP made intellectual contributions. All authors contributed to the article and approved the submitted version.

## Conflict of Interest

The authors declare that the research was conducted in the absence of any commercial or financial relationships that could be construed as a potential conflict of interest.
