# Supplementary material for: Increased Neutrophil Count and Decreased Neutrophil CD15 Expression Correlate With TB Disease Severity and Treatment Response Irrespective of HIV Co-infection
Source: Front Immunol. 2020 Aug 28;11:1872. doi: 10.3389/fimmu.2020.01872 (PMC7485225; doi:10.3389/fimmu.2020.01872)
Supplement: Supplementary file 1 [file Data_Sheet_1.PDF]

Chest X-Ray

Participant ID	______________ (031-ss-nnnn)
Demographic Information	
Date of Chest X-Ray	______________
Date of Birth	______________
Age	______________
Gender	◯ Male ◯ Female
Chest X-ray not done pregnant	☐
Chest X-ray not done other reason	☐
Chest X-Ray Findings	

Right Lung Opacity

	Cavitation	Opacity	No opacity
Lung Opacity (Shadows) right upper	☐	☐	☐
Lung Opacity (Shadows) right middle	☐	☐	☐
Lung Opacity (Shadows) right lower	☐	☐	☐

Right Lung Pathological Findings

	Present	Absent
Mediastinal adenopathy right	◯	◯
Pleural effusion right	◯	◯
Hilar adenopathy right	◯	◯
Bronchiectasis right	◯	◯
Collapsed lung right	◯	◯

Percentage of lung affected (Right Lung)	______________ (%)

Other chest xray finding specify

______________________ (right lung)

Left Lung Opacity

	Cavitation	Opacity	No opacity
Lung Opacity (Shadows) left upper	☐	☐	☐
Lung Opacity (Shadows) left middle	☐	☐	☐
Lung Opacity (Shadows) left lower	☐	☐	☐

Left Lung Pathological Findings

	Present	Absent
Mediastinal adenopathy left	◯	◯
Pleural effusion left	◯	◯
Hilar adenopathy left	◯	◯
Bronchiectasis left	◯	◯
Collapsed lung left	◯	◯

Other chest xray finding specify

______________________ (left lung)

Percentage of lung affected (Left Lung)

______________________ (%)

Overall Lung Findings

Total Percentage of lung affected

______________________ (%)

Is cavitation present?

◯ Yes
◯ No
◯ Not applicable
◯ Unknown

Cavitation Score

______________________ (points)

Chest X-ray Score

______________________ (points)

Other chest xray finding specify

______________________ (all lungs)

Name of chest x-ray reader recorded

____

Date read

____
