# Supplementary material for: Increased Neutrophil Count and Decreased Neutrophil CD15 Expression Correlate With TB Disease Severity and Treatment Response Irrespective of HIV Co-infection
Source: Front Immunol. 2020 Aug 28;11:1872. doi: 10.3389/fimmu.2020.01872 (PMC7485225; doi:10.3389/fimmu.2020.01872)
Supplement: Supplementary file 2 [file Table_1.DOCX]

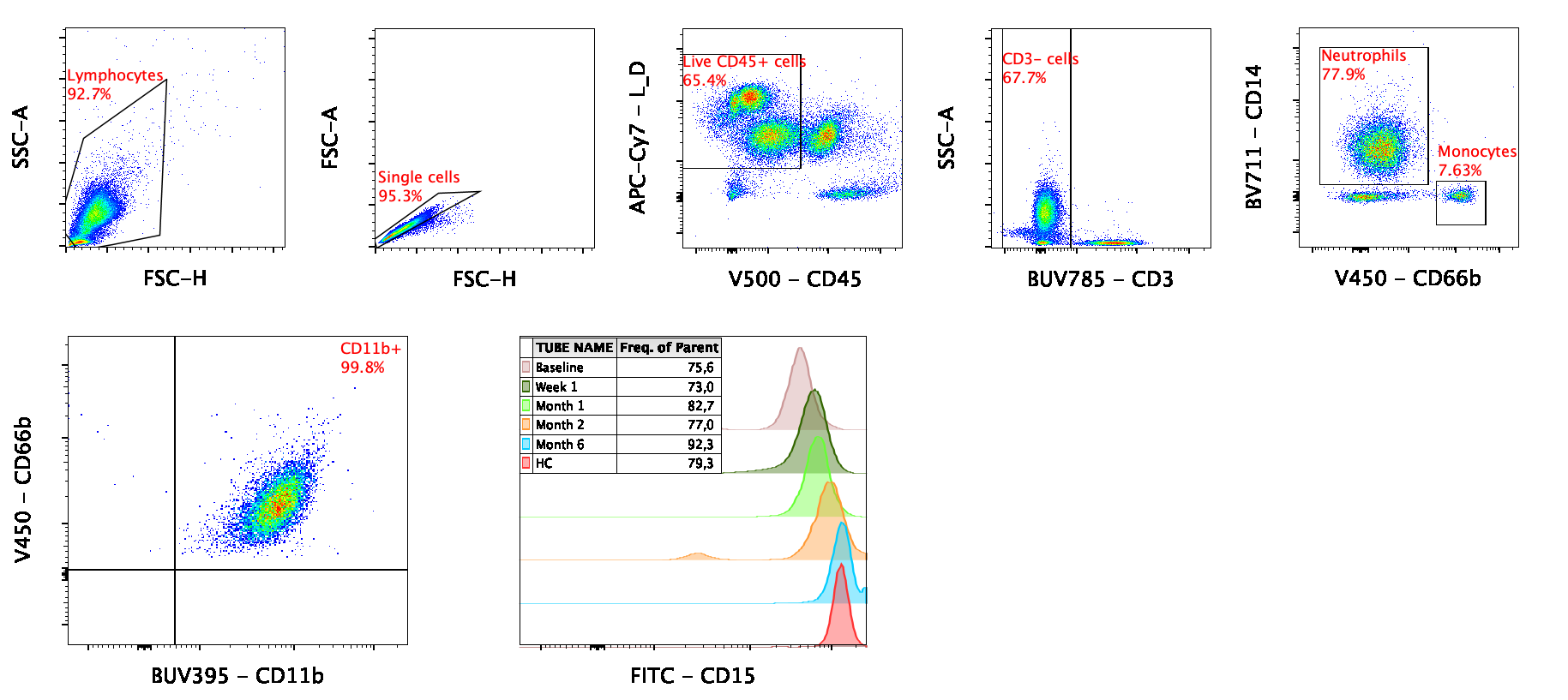


**Figure 1. FASC analysis of neutrophils**. Neutrophils were stained for flow cytometry and analyzed on the FACSAria Fusion. Figure **A** represents that standard gating strategy with neutrophils identified as CD66b^+^ cells. The numbers are percentages of cells falling in each gate. **(B)** All neutrophils are CD11b^+^. **(C)** Histograms representing change in CD15 MFI observed in TB cases from baseline/pre-treatment to treatment completion compared to healthy controls (HC).

**Figure 2: Effect of HIV status on baseline bacterial load and Chest X-ray scores.** Box plots of **(A)** baseline bacterial load and (**B)** CXR scores among HIV negative and positive participants. Data represents median values and was analysed using Mann-Whitney t-test. **(C-E)** Spearman’s rank correlation analysis of CD4 T cell counts with bacterial burden and CXR scores.

**Figure 3: Longitudinal measurement of blood neutrophil count, NLR ratio and CD15 expression during anti-TB treatment.** Changes in neutrophil blood (PMN) count **(A),** PMN/Lymphocyte ratio **(B)** and surface CD15 expression **(C)** levels over the course of treatment in TB cases stratified by HIV infection. P-values were obtained using the Wilcoxon rank sum test and Kruskal-Wallis tests for multiple comparisons. Dotted line represents the median healthy control levels.

**Figure 4: Neutrophil blood counts and CD15 expression correlate with bacterial burden at week 3 post-treatment initiation.** Spearman’s rank correlation analysis of neutrophil blood (PMN) counts and CD15 expression with bacterial burden **(A-C).**
